# Mathematics and statistics distance learning: more than just online teaching

**DOI:** 10.1093/teamat/hrab012

**Published:** 2021-09-06

**Authors:** Rachel Hilliam, Derek Goldrei, Gaynor Arrowsmith, Alexander Siddons, Cath Brown

**Affiliations:** School of Mathematics and Statistics, The Open University, Milton Keynes MK7 6AA, UK

## Abstract

At the Open University, where students learn online and at a distance, the School of Mathematics and Statistics has for many years provided innovative ways of supporting students outside the ‘classroom’ environment so was well prepared to support students during the COVID-19 pandemic. These forms of support include online forums to help students with module choice and taster resources including diagnostic quizzes for students to self-assess their readiness to study individual modules and receive targeted support. Since 2017, these resources, and more, have been incorporated into a multi-functional student-facing website. The website enables all units, both academic and non-academic, to provide consistent academic, pastoral and social support to students studying mathematics and statistics modules online. By focusing on the different stages of a student’s journey, the website provides a one-stop shop for students to self-serve and obtain appropriate support at each point in their own student lifecycle. Data gathered on the frequency of use of the website, together with the results from staff and student questionnaires, have provided insight into how students and staff use the website. The evaluation highlights the need for clear signposting to such resources. In addition, the wide range of resources which enable students to make informed module choices is shown to be particularly important for staff who provide pastoral and academic support to students.

## 1 Introduction

Providing a coherent student experience is complex and multi-facetted, and the literature regarding student support spreads across many differing areas of support. For example, there have been investigations into the social support students require as they transition to university and the need for students to integrate into peer groups (Wilcox *et al*., 2006), while other studies consider the pastoral support which student require (McChlery & Wilkie, 2009). However, according to Carlson *et al.* (2016), the category of student support which still receives the greatest attention in the literature is academic support. Nevertheless, cultural changes have taken place in universities in recent years with the result of a shift in focus from purely academic support to wider social and pastoral student support delivered through coherent and effective student-facing activities (Temple *et al*., 2014). Stodt & Klepper (1987) identified the importance of collaboration between academic departments and a range of non-academic units in order to provide effective academic, social and pastoral support.

To help practitioners improve the student experience across every stage of the student lifecycle, Morgan (2012) sets out the student experience practitioner model. This model identifies six stages of the study lifecycle:

- First contact and admissions
- Pre-arrival
- Arrival and orientation
- Introduction to study
- Reorientation and reinduction
- Outduction

Morgan (2012) argues that at each stage of the lifecycle, if students are to successfully complete their studies, they need appropriate advice, support and guidance which can be placed into five linked themes:

- Curriculum and assessment
- Pedagogy
- Support
- Finance
- Employment

For example in the student experience practitioner model, at the *First contact and admissions* stage, students may need information on the following: which subjects are studied in a degree and what type of assessments are undertaken each year (curriculum and assessment), a summary of the different teaching and learning methods that are used (pedagogy), an overview of the range of support units and facilities available (support), information on loans and cost of studying (finance) and support on working while studying and placement options (employment). The type of support needed at each stage of the lifecycle will change, but to deliver this comprehensive student experience, all staff from across the institution need to be actively involved in the initiatives and activities.

While all the stages of the lifecycle in the student experience practitioner model are important, there are specific challenges for students in the early stages who transition to university to study mathematics and statistics (Pell & Croft, 2008). These challenges exist both for students who choose to study a mathematics and statistics qualification and for students who will require mathematics and statistics skills while studying a qualification from another discipline, for example, economics, science or engineering. The transition support for the mathematics and statistics qualification students is typically provided by the mathematics and statistics department or school who teach and manage those degrees. In contrast, the mathematics and statistics support which students studying other disciplines may require is often provided through dedicated mathematics and statistics support centres (Grove *et al.*, 2020).

The transition to university for mathematics and statistics students is challenging for many universities who need to cater for students who come from a range of different backgrounds and prior mathematical experience. Often universities use a diagnostic quiz on entry to check the mathematical level of understanding. A report by the UK Engineering Council (2000) found at least 60 Departments of Mathematics, Physics and Engineering gave diagnostic tests in mathematics to their new undergraduates. One of the nine findings within the report (p. iii) states *‘Diagnostic testing should be seen as part of a two-stage process. Prompt and effective follow-up is essential to deal both with individual weaknesses and those of the whole cohort.’* Many universities have taken this advice and a study at the Lincoln School of Engineering showed how the results from a diagnostic test could be used to devise individual learning plans for students resulting in an increase in retention (Gallimore & Stewart, 2014). Even if students have all the prior mathematical knowledge they are assumed to need, the transition to learning mathematics at university level can be daunting as there is a change of emphasis between school and university mathematical learning activities (Jooganah & Williams, 2016). The breadth of support that students may need is likely to cover all the themes in Morgan’s model and diagnostic quizzes provide one way of identifying what support is needed.

Mathematics and statistics support centres provide vital support for students who may struggle with mathematics and statistics which students require to study their chosen discipline successfully. A large and comprehensive survey in 2018 showed that at least 75% of institutions which participated in the survey provided some form of mathematics support centre for use by students studying a wide and expanding number of subjects (Grove *et al*., 2020). These centres provide not only support to plug gaps in students’ knowledge but also build students’ confidence (Warwick, 2010).

At the Open University (OU), where students learn online and at a distance, the School of Mathematics and Statistics (M&S) provides learning opportunities for students across a wide range of qualifications. This paper will outline how the School of M&S has worked with non-academic units to create a website, which acts in part as a repository of support materials, to enable consistent academic and pastoral support to be provided for all students studying M&S modules regardless of their chosen qualification. In addition, the website includes a forum to which students and all academic and non-academic units contribute, hence providing the social support which is so important to learning mathematics and statistics.

## 2 Teaching mathematics and statistics at the OU

The OU is one of the largest universities in Europe, providing distance learning education to over 150,000 students. It is also the largest provider of higher education to students in the UK who have a declared disability with 27,237 such students in 2018/19 (The Open University, 2020). Having spent 50 years developing and delivering distance learning modules, the OU has a world-wide reputation built on its distance learning methodology. Since its inception in 1969, the OU has continually updated its practices to incorporate developments in both pedagogic theory and technology (Weinbren, 2014).

Students at the OU engage with distance learning through a combination of high-quality teaching material (both printed and online) and receive correspondence tuition provided by a network of about 6,000 tutors officially designated associate lecturers (ALs). Several ALs are contracted to each module and each supports a group of students, usually 20, through their study of that particular module. The ALs provide distance learning events (where the AL and students can converse, these are usually recorded for later viewing), correspondence tuition (via feedback on continuous assessment) and one-to-one academic support via email and telephone. Because ALs only provide academic support for individual modules, students are not able to access an AL for ongoing support between module study. This means that in Morgan’s lifecycle ALs can offer support (academic, some pastoral and social) only for the *Introduction to study* and *Reorientation and reinduction* stages of the module they support students through.

The ongoing pastoral support is provided by staff in one of the four student recruitment and support centres (SRSCs). Each SRSC is linked to one of the four faculties in the University and provides financial and fee advice, administration and registration advice, assessment of prior learning and credit transfer, induction to the University and exit and career advice. The SRSC staff cover Morgan’s themes of finance, employment and support of a pastoral kind. In addition, these centres provide support for study planning by students. This can take a variety of forms, such as students needing to take a break from study or needing specialist additional support due to a disability or wanting information to help with module and qualification study planning. This type of support crosses the areas of pastoral and academic support and therefore will often require academic knowledge about the modules and qualifications. This more specialist information and guidance are provided by a dedicated student support team (SST), who are part of each SRSC and who have responsibility for providing such support to students studying individual disciplines or subjects. While the SST staff are dedicated to one discipline area, they do not have academic knowledge of that curriculum and therefore each SST works with the appropriate academic school to ensure the correct academic support is provided to each student.

The School of M&S resides in the Faculty of Science, Technology, Engineering and Mathematics (STEM). The associated STEM SRSC includes four subject SSTs: M&S, computing and communications (C&C), engineering and innovation (E&I) and Science. Each SST has two types of advisory staff:

- Senior advisors who work in an individual SST giving advice. This is defined as giving information plus further exploration to help students understand information in the context of their circumstances and abilities. (At the time of writing, there are seven senior advisors in the M&S SST.)
- Educational advisors who work in an individual SST giving guidance. This is defined as giving information and advice plus further exploration of students’ personal and/or emotional issues that may prevent barriers to study. This work can involve sensitive challenging of student perceptions and/or advocating on their behalf. (At the time of writing, there are two educational advisors in the M&S SST.)

While each of the senior and educational advisors are linked to just one of the four SSTs, at busy times they help out across the entire SRSC. In addition to the SST staff, advisors based in student recruitment and fees (SRF) work across the entire SRSC. These advisors are front-line staff who do not specialize in the curriculum but provide more general information to students, defined as gathering and providing information, together with encouraging and facilitating students to research available information.

The School of M&S has built up a strong working relationship with the M&S SST. This means that both the academics in the School of M&S and the M&S SST staff work together to ensure students receive the appropriate academic, pastoral and social support at each stage of their lifecycle. This partnership model has resulted in several initiatives to the student experience for M&S students (Hilliam & Williams, 2019). One such initiative is the use of diagnostic quizzes described in Section 3.1.

The OU, like many universities, provides M&S teaching for students who study these subjects as part of non-mathematics and statistics qualifications. Many of the M&S modules are simultaneously studied by students on multiple different qualifications. For example, there were roughly 1500 students studying the large level 1 mathematics module *Essential Mathematics I* (MST124), in the 2019 September cohort. Of these students, less than half were studying for a qualification in M&S. The rest were mainly studying for a qualification in a wide range of disciplines including computing & IT, cyber security, natural sciences, economics and engineering, together with 110 students who were studying the module in isolation (that is, not linked to any qualification). This presents several challenges when it comes to supporting students on modules such as MST124. The academic support needed is often different for non-mathematics and statistics students. For such students, it is often important to develop their confidence and belief in their mathematics ability. This support is provided largely by each student’s assigned AL.

The pastoral support is slightly more complicated as non-M&S students are supported by their own SST: for example, IT students will be supported by the C&C SST rather than the M&S SST. It is therefore important that students receive consistent advice, regardless of which SST they are talking to. The School of M&S worked closely with the M&S SST to develop resources which would assist all SST staff to provide appropriate information, advice and guidance to students studying M&S modules.

In 2017, study sites for each subject area were introduced by the university with the aim that these websites would complement individual module websites and provide a home website for students throughout their qualification. While students register for a qualification, they only have access to the individual module websites while they are studying each individual module. Each module website provides distance learning material including interactive quizzes, videos, computer animations and online forums where students can interact with their peers and academics. However, when a module finishes, the students no longer have access to these forums and therefore are left without any official peer or academic support. The study sites were designed to fill this gap.

The School of M&S took this opportunity to create a one-stop shop to support the lifecycle of the student, following the stages of Morgan’s practitioner model (Hilliam and Arrowsmith, 2019), and consolidate resources developed by the School of M&S and M&S SST into the M&S study site. The aim was to provide a coherent set of resources which all students, ALs, SST and SRF staff could use for any M&S related query regardless of the actual qualification which the student was studying. This paper will outline the approach taken to create the website and evaluate whether it was effective in the aim of providing the necessary resources for ALs, SST and SRF staff to support all students who are studying M&S modules.

## 3 The M&S study site

**Fig. 1.**
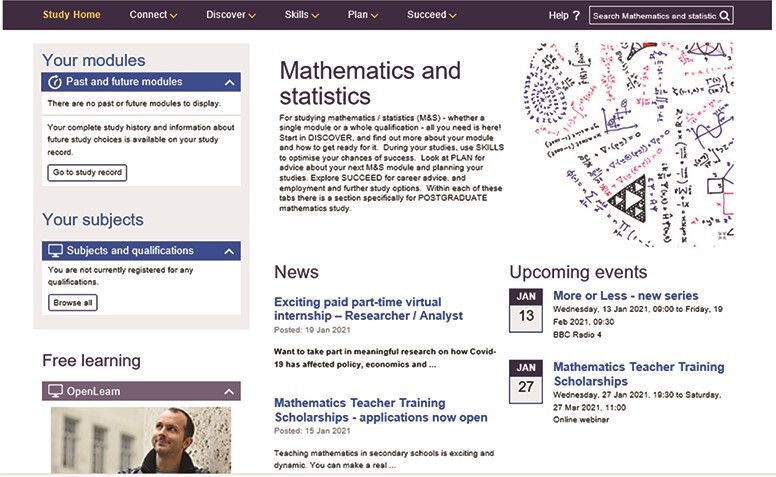
The M&S study site.

There is a generic layout for all study sites at the OU which are split into six sections: Study Home, Connect, Discover, Skills, Plan and Succeed. While Study Home shown in Fig. 1 conforms to a standard template, the content that populates the remaining sections is at the discretion of each curriculum area.

The M&S study site is hosted on the OU’s virtual learning environment (VLE) which enables the university to monitor the way in which students use the site. As students must already be registered with the OU before they get access to the study site, the first stage of Morgan’s lifecycle, that of *First contact and admissions*, is dealt with outside of the study site. Some of the M&S resources from the study site, such as the diagnostic quizzes and preparatory material, are repeated on a website (OpenMS) that is open for anyone to view with the aim of assisting prospective OU students make an informed choice https://learn1.open.ac.uk/course/view.php?name=OPENMS. Once students are registered with the OU they have access to the study site and will use the resources directly from the study site. While the ordering of the tabs on the study site do not directly link to Morgan’s lifecycle, the progression from Discover through to Succeed roughly follows the stages in the lifecycle from *Pre-arrival* to *Outduction* as outlined in the following subsections.

### 3.1 Discover

The Discover section of the website covers support for the *Pre-arrival* and *Arrival and orientation* stage, by providing short videos on a range of topics such as what it is like to study M&S with the OU. Students on most of the M&S qualifications can start their studies with one of two possible modules. The diagnostic quiz, named *Are You Ready For (AYRF)* MST124, enables students to self-assess their readiness to start with *Discovering Mathematics* (MU123) or the more advanced *Essential mathematics I* (MST124). This *AYRF* MST124 quiz is also available in the OpenMS website and accessible to anyone outside of the OU. The M&S SST uses the recorded quiz attempts to give appropriate advice and guidance, which has an enormous impact on retention on the module (Calvert *et al*., 2016). Since, as was noted in Section 2, MST124 serves multiple qualifications, not just M&S qualifications, the *AYRF* MST124 is particularly important. The OU has an open-entry policy for its qualifications. Therefore, the range of prior mathematical ability of students is considerable. For many students, it may have been several years since they have formally studied any mathematics, so assessing their readiness to study MST124 is vital if they are to succeed. Together with the result prompting a conversation with the SST, feedback and further resources are provided based on the student’s *AYRF* answers. This is one example of the M&S study site not only being the repository for information which the student can access but providing the mechanism by which integrated academic and pastoral support can be provided. In 2014, a survey into the learning and teaching experiences of students, carried out by the Higher Education Policy Institute in conjunction with the Higher Education Academy, showed that one in four first-year students found information provided by institutions to be ‘vague’ (Soilemetzidis *et al.*, 2014). In addition, Diamond *et al*. (2014) state that in order to provide students with adequate support, information must be complemented by sufficient advice and guidance. The M&S study site strives to address both points by ensuring the information regarding module choice is explicit and integrated support is provided for students, for example by using the results from the *AYRF* quiz.

### 3.2 Skills

The Skills section covers the *Introduction to study* stage of the student lifecycle, with resources covering a range of study skills specific to M&S, which include the following:

- How to study mathematics and statistics effectively
- Problem solving
- Writing mathematics and statistics
- Mathematical proof
- Understanding different forms of writing: description, analysis, reflection
- Typing mathematical notation
- Software used in M&S modules

with further plans for resources on ‘Mathematical modelling’ and ‘Working with data’.

A recommendation from the Higher Education: Retention and Engagement report was to provide good communication about additional student support (Foster *et al.*, 2012). To achieve this, there is a dedicated section on overcoming accessibility issues when studying M&S which contains detailed information for students who need additional resources and alternative formats for their study material. These resources are useful for the SSTs to use in conversations with students who have declared disabilities and who may need both specialist academic and pastoral support.

### 3.3 Plan

The Plan section has resources for people who are ready to register for their next M&S module, so are at the *Reorientation and reinduction* stage of the lifecycle. There is a collated table of the last four years of pass and completion rates for each M&S module and a further table giving the last four years of student feedback from in-house student-satisfaction module surveys. There is also information about new modules that will be available in future years, changes to existing qualifications and any new qualifications that are being planned. One of the most popular set of resources is the *Discover your module* (*DYM*) subpages where students can get a taste of each module. Each module’s *DYM* page has a module syllabus, outline calendar, the first four to six weeks of learning material (modules typically have 30 weeks of learning material) and its own dedicated *AYRF* quiz. These quizzes take a variety of forms and in many cases are of a similar format to the quizzes which form part of the assessment on many of the M&S modules (Lowe, 2015). The *DYM* pages are also available from the Discover section of the website, as it is possible for OU students to study a single module without it being part of any qualification.

**Fig. 2.**
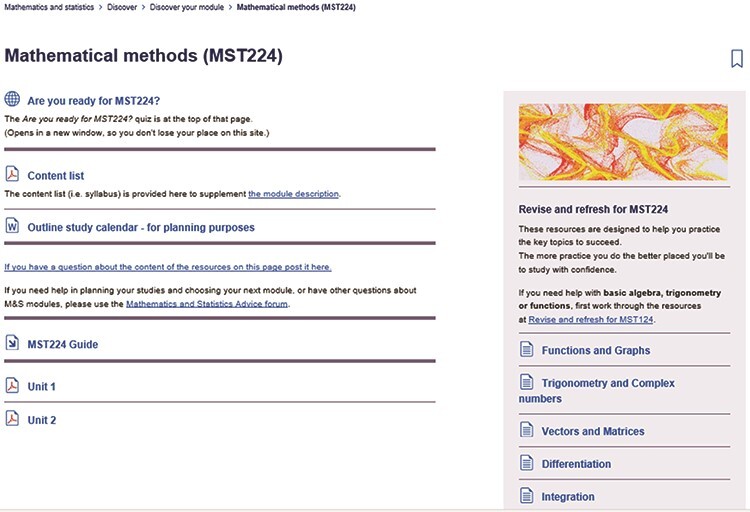
*DYM* subpage for a second-level mathematics module, *Mathematical Methods*, MST224.

Students who study with the OU not only come with a variety of pre-undergraduate mathematical knowledge: the vast majority also have other work and caring responsibilities alongside their study. This can mean that some students can take many years to finish their degrees and have long gaps between periods of study which may result in loss of the ability to use various mathematical techniques. When the students are studying a module, they have access to an AL who can give them help and advice and forums where they can discuss problems with other students. Between modules and during any study breaks, students do not have an assigned AL and contact, so there is limited help available. To alleviate this issue, the School of M&S has a suite of distance learning support sites together with dedicated forums which can be used by students to help them prepare for their next module: these are named *Revise and Refresh* (*R&R*). The *R&R* resources are available in a panel on the right of each *DYM* subpage within the Plan section (Fig. 2). This material takes the place of an online mathematics support centre, where students can brush up on their knowledge, identify any gaps and have access to academic support (Pawley & Hughes, 2018). It also enables students to test whether the module they are planning to take next is at the correct level for them, given their current ability level.

Some modules also provide a way of allowing students to use the gap between modules to make an early start on their next module. One example is the *Early start* programme on a large level 1 statistics module, *Introducing Statistics*, M140, taken by roughly 1,500 students per year. The *Early start* programme, which successfully improved retention, offers AL-led support for three months before the module starts and access to the statistics software used on the module (Calvert & Hilliam, 2018). Several modules have a similar provision, and these are accessed through the appropriate *DYM* subpage.

### 3.4 Succeed

The Succeed section covers the last stage of the lifecycle that of *Outduction*. This section contains resources on careers and employability in M&S: what employers are looking for and how to articulate this in applications, ways in which students can enhance their employability and advice and guidance on job-seeking and making an application. The resources have been jointly produced by the School of M&S and the central OU Careers Service, together with external support from the Institute of Mathematics and its Applications and the Royal Statistical Society. There is also advice on studying beyond an undergraduate degree.

### 3.5 Connect

The sections from Discover through to Succeed aim to provide a one-stop shop to help students choose their next module, use the time between module study to revise pre-requisite knowledge and make a head start, give advice about careers and link to ways of receiving academic and pastoral support. However, it is necessary to also provide a means for students to receive social support. One way of providing a mechanism for social support is through the module advice forum found under the Connect tab. This forum is used by students to discuss questions around module choice and it enables the entire OU M&S community to engage in conversations throughout the entire year, rather than students only being able to communicate with students on the same module while studying that module. While the focus of the forum is on module choice, it also provides a place for students to feedback on other issues and there is active engagement from students, ALs, academics and SST staff (Hilliam & Goldrei, 2019). The forum enables students to act as both the learner and the instructor as they progress through their studies, with staff engagement to guide the discussion and resolve any conflicts. With the range of contributors across academics, ALs, SST staff and students, the result is a forum which provides academic, pastoral and social support.

While the resources described in Sections 3.1–3.5 are for undergraduate study, similar resources exist for postgraduate M&S students.

## 4 Evaluation

The M&S study site went live to students on 19 September 2017. While some initiatives such as the *AYRF* MST124 quiz were already in use prior to this date, the creation of the study site saw the coordination and assimilation into one place of many of these resources. The close working relationship between the School of M&S and the M&S SST had ensured that the resources were heavily used by M&S SST staff during conversations with students regarding module choice and study planning. What was less clear was how the resources were used by students, ALs, non-M&S SST and SRF staff.

As the website is embedded in the VLE, it is possible to track the number of unique student visits to the website and the total number of visits in any given period. These data can therefore be used to evaluate when the website is used and how often. Evaluation was therefore carried out to ascertain how heavily the site was used by students. In addition, two questionnaires were distributed: one to students and one to staff. The aim of the questionnaires was to collate feedback on how people used and found out about the study site, together with collating suggested improvements. In addition, there were questions regarding how students used the forums embedded in the study site and whether these forums together with various online and face-to-face events created a sense of community among mathematics and statistics students. This paper will only concentrate on the feedback regarding how students and staff find and use the study site, and the resources designed to help students choose the appropriate module to study. Hilliam (2020) provides an evaluation of the whole project.

### 4.1 Study site usage

As noted in Section 3, students study M&S modules as part of a wide range of qualifications. Since each discipline area has its own study site, the ‘home’ study site for, say, an economics student is not the M&S study site but the equivalent economics study site, even if that economics student is currently studying an M&S module. This has implications for evaluation as only the data on visits to the M&S study site by students studying for M&S qualifications are stored in the VLE. This is unfortunate when evaluating the effectiveness of the M&S study site since many of the resources are particularly designed for students studying a range of qualifications who need to assess their mathematics ability to check that are prepared to study their chosen M&S module. To give an idea of the scope of this problem in 2019/20, the number of students studying an M&S qualification was 7947, whereas the number of students studying an M&S module as part of another qualification was 7683. Therefore, the VLE data available only provide information on about half the students who potentially could benefit from the website. While analysing the usage is still useful, it must be interpreted in the knowledge that resulting conclusions are based on biased information.

**Fig. 3.**
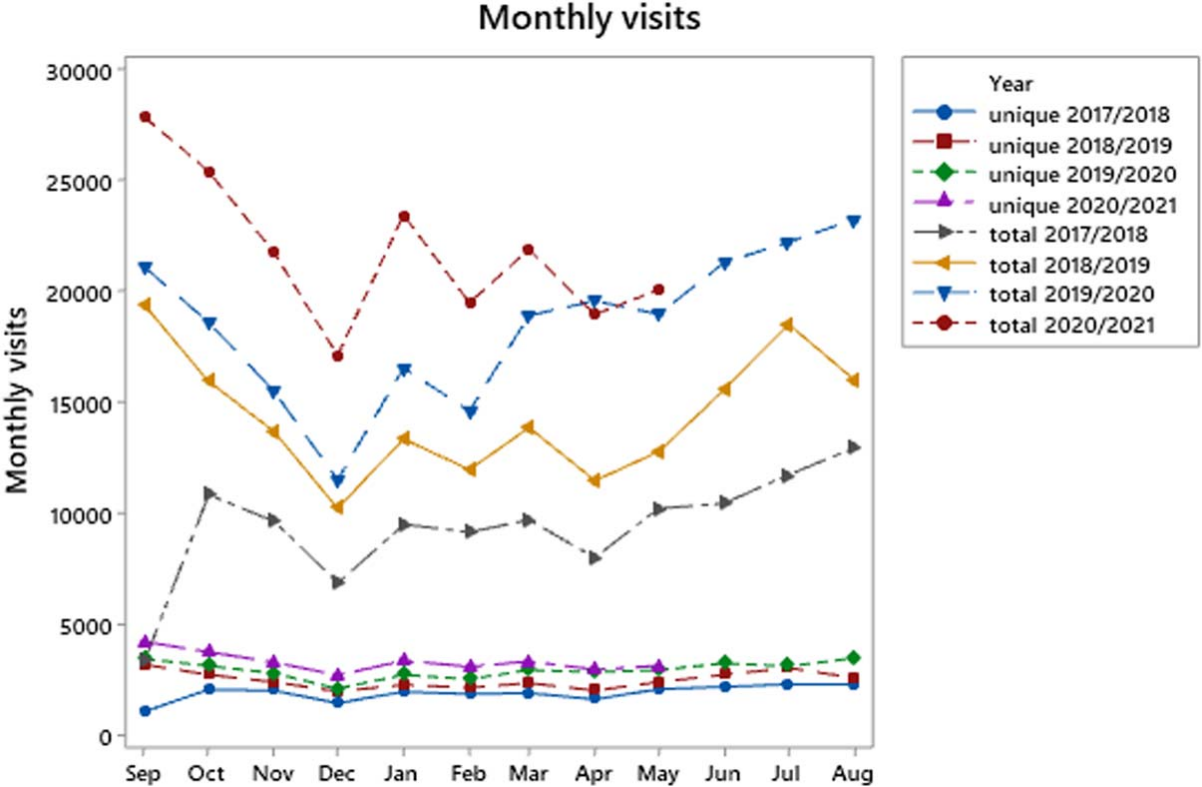
Unique and total number of monthly visits to the M&S study site for months September 2017 to May 2021.

Roughly half of the students, who are linked to an M&S qualification, use the site each month. The lower four lines in Fig. 3 show the number of different students who visit the site each month: this is referred to as the number of unique monthly visits, while the upper four lines show the total number of monthly visits as each individual student may visit the site multiple times each month. The majority of OU students study a module from September through to the following June. The annual pattern of visits to the subject site peaks in September when individual module websites open (all M&S module websites have a link to the M&S study site) and in June/July when most module results are released (Fig. 3). There are dips around the holiday periods of Christmas and Easter each year. While less than half of the students who are linked to an M&S qualification appear to use the site, it is clear that once students find the site they return to it on a frequent basis.

Figure 3 shows an overall increase in the number of students using the study site year on year. Although only half of the M&S students appear to use the study site, early feedback from consultations with students in 2018 suggested that students had problems finding the study site. In order to signpost the study site more clearly, several initiatives were put in place during 2019/2020 including the following: the creation of a student newsletter which highlighted the study site, providing an outline of the study site on the back of the UG module contents checklist mailed out to students with their print texts, inclusion of website links in general OU communications, reinforcement by SST individual contact and a series of emails, particularly aimed at newly registered UG students pointing towards the *DYM* pages. Figure 3 shows a growth in the number of students who use the study site with the largest increase for the year starting in September 2020, each initiative appears to have a commulative effect on the number of students reaching the study site.

### 4.2 Student questionnaire

As the data in Subsection 4.1 are only available for students studying an M&S qualification, it is important to collect information regarding how all students, including students who study an M&S module as part of a non-M&S qualification, find the study site and evaluate which resources they find useful. A questionnaire was constructed in, and administered though, Jisc online surveys. The questionnaire opened on 15 June 2020 to a sample of 500 students. The sample consisted of the following:

- 250 students on an undergraduate qualification in M&S,
- 50 students on a postgraduate qualification in M&S,
- 100 students on a non-M&S qualification (for example, computing, economics and science) but studying an M&S module,
- 100 students studying an M&S module not linked to any qualification.

There were 68 respondents giving a response rate of 14%. Of the respondents, 38 were studying for an M&S qualification and 30 studying for a non-M&S qualification, which is roughly equivalent to the population proportion of students studying M&S versus non-M&S qualifications. The results presented below are a subset of the questions and responses; more detail can be accessed in the internal project report (Hilliam, 2020).

While 73% of respondents had used the study site, there is a difference between the proportions of M&S qualification students who use the site (84%) compared to non-M&S qualification students (60%) (Table 1).

**Table 1:** Student responses to the question: have you used the M&S study site?

	M&S	Non-M&S	Total
Yes	32 (84%)	18 (60%)	50 (73%)
No	6 (16%)	12 (40%)	18 (27%)
Total	38	30	68

The 18 respondents who had not used the study site were asked to choose a reason why they had not used the site. A high proportion, 67%, of non-M&S respondents did not realize the study site was there, compared to only 17% of M&S respondents. The main reason M&S respondents gave for not using the site was that they had not needed to use it (Table 2). These findings are consistent with a large study on Mathematics Learning Support in Ireland, where when asked about using mathematics support ‘weaker’ students were more likely to say they ‘hadn’t heard of it’ whereas ‘stronger’ students responded that they did not need it but would use it if they did (O’Sullivan *et al*., 2014). Lawson (2015) discusses that there are often deeper reasons for such responses by students. However, in the case of the M&S study site, it is entirely possible that non-M&S students are unaware of the M&S study site, due to how students are directed to home study sites, rather than the study site linked to the module they are currently studying. Therefore, more work is required to point out the benefits of different areas of the site to all students.

**Table 2:** Student responses to the question: if you have not used the M&S study site, please state why not

	M&S	Non-M&S	Total
Did not realize it was there	1(17%)	8 (67%)	9 (50%)
Have not needed to use it	5 (83%)	1 (8%)	6 (33%)
Have not had time to engage with it	0 (0%)	3 (25%)	3 (17%)
Total	6	12	18

Respondents were given the fixed options listed in Table 3, regarding how they were made aware of the site and could select more than one option. Most respondents found the study site from either a link on their module webpage or a link from StudentHome, which is the landing page when students log into the OU system. However, the latter is considerably smaller for non-M&S students as there is no obvious way to link to the M&S study site from StudentHome if students are studying for a non-M&S qualification. Feedback from student consultations has shown that one of the most effective ways of pointing students to the study site is from direct email contact with the OU regarding studies. This may well explain why there is such an increase in the number of students using the M&S study site in September 2020 (Table 3) as there had been several emails pointing students to the study site during July and August. It is therefore important that all communications to students regarding their study should contain links to appropriate areas of the study site.

**Table 3:** Student responses to the question: how were you made aware of the site?

	M&S	Non-M&S	Total
Link from StudentHome webpage	25	12	39
Link from module webpage	19	11	31
Email from the OU regarding your studies	7	6	13
Information on content checklist sent with your M&S module books	3	6	9
Link from ‘New to OU study’ induction website	3	4	7
Discussion with the SST	1	5	6
Discussion with a tutor	1	4	5
Mathematics and statistics newsletter	2	2	4
Discussion with a fellow student	1	2	3
Information on a card about thestudy site given out at an event	0	0	0

When asked how students used the study site, respondents were given the fixed options listed in Table 4 and could select more than one option. The most popular responses were to help students to choose their next module, make a head start and revise and refresh their knowledge.

**Table 4:** Student responses to the question: how do you use the study site?

	M&S	Non-M&S	Total
To get a head start	24	12	36
Help when choosing next module	21	12	33
To revise and refresh	19	7	26
Browsing	12	6	21
Connect with other M&S students or staff	9	6	15
To get careers advice	4	2	6
Info about external M&S societies	2	4	6

General feedback from students about the study site focussed on the difficulty with navigating around the study site and finding the site, though when students do find the study site, qualitative feedback suggests they particularly appreciate being able to get early access to module material.

*The resources are great: still it’s still quite difficult to find some things, to find say the Revise and Refresh material still takes quite a bit of hunting.*

*I have found it very helpful for accessing addition information about possible modules I can study than is provided by the general module descriptions, as well as accessing materials to get started with the module, which has been very useful.*

### 4.3 Staff questionnaire

One of the purposes of the study site was to help students with module choice. Therefore, it is important that all staff who support students through conversations around module choice are aware of the resources on the study site. To explore how different staff use the study site, an email invitation to complete a questionnaire, in Jisc, was sent to all ALs (roughly 300) who tutor on an M&S module and 42 SST staff (12 STEM educational advisors and 30 STEM senior advisors) on 22 June. Due to unprecedented call volumes, it was not possible for SRF staff (advisors) to compete the questionnaire during this period; therefore, a copy was made available to the 60 SRF staff linked to STEM on 27 October. There were 135 responses to the staff questionnaire: 65 from ALs (22% response rate), 32 from SST staff (76% response rate) and 38 from SRF staff (63% response rate).

The majority (93%) of SST staff respondents but only 56% of AL respondents had used the study site (Table 5). When the study site was created in 2017, one of the aims was to provide an area with readily available resources which SST staff could point students towards. It was therefore particularly gratifying to see the large proportion of SST staff respondents who had used the site as this number included non-M&S SST staff.

**Table 5:** Staff responses to the question: have you used the M&S study site?

	AL	SST	SRF	Total
Yes	36 (56%)	29 (94%)	13 (34%)	78 (59%)
No	28 (44%)	2 (6%)	25 (66%)	55 (41%)
Total	64	31	38	133

Respondents were given the fixed options listed in Table 6 when asked why they used the site and could select more than one option. The main use of the study site by SST staff respondents who had used the site is to direct students towards resources and to enhance their conversations with students, though several staff also use the site for their own information. The majority (12 out of 13) SRF staff respondents who use the site do so to point students towards resources. A disappointingly low number of SRF respondents use the site to aid their conversations with students. This is of particular concern as these staff are the first point of contact (rather than the student’s individual AL) when a student phones the OU for help. The majority of the 36 AL respondents who use the site use it both for their own information and to direct students towards the resources.

**Table 6:** Staff responses to the question: why do you use the site?

	AL	SST	SRF	Total
To point students towards resources	30	28	12	70
For my own information	32	16	4	52
To aid conversations with students	17	27	6	50
To link up with other M&S staff and/or students	3	1	0	4
Number answering this question	36	29	13	78

The respondents who had not used the study site were given the fixed options listed in Table 7 and could select more than one option. Most AL respondents either did not know the study site existed or had not found a use for it. Over half of the SRF respondents who did not use the study site did not know the site existed. It is a concern that nine members of SRF respondents have not found a need to use it, given that there are a large number of resources on the site to help with module choice. More positively, there were only two SST respondents who had not used the site. Interrogating the data more closely revealed that the one who did not realize it existed was a senior advisor in the E&I SST and the one who had not had time to engage with this site was a senior advisor in C&C; all staff who are linked to the M&S SST knew about the study site and had used it.

**Table 7:** Staff responses to the question: why have you not used the M&S study site?

	AL	SST	SRF	Total
Did not realize it was there	17	1	19	37
Have not found a need for it	14	0	9	23
Do not have the time to engage with it	6	1	4	11
Did not know how to find it	5	0	3	8
Number answering this question	28	2	25	55

All the staff respondents (even those that had not yet used the site) were asked, using a Likert scale, to rate how useful they found various resources, of particular interest are the *AYRF* quizzes, *DYM* pages and the M&S advice forum. Most of the respondents found these resources either quite useful or very useful (Table 8).

**Table 8:** Staff responses to the question: how would you rate each of the following resources for use by you or your students?

	Not at all useful				Only slightly useful				Quite useful				Very useful			
	AL	SST	SRF	Total	AL	SST	SRF	Total	AL	SST	SRF	Total	AL	SST	SRF	Total
*AYRF* quizzes	1	0	0	1	3	0	0	3	15	0	9	24	34	30	30	94
*DYM* pages	1	0	0	1	3	0	1	4	13	3	10	26	35	27	23	85
M&S advice forum	1	0	0	1	2	3	4	9	26	12	20	58	21	12	9	42

A number of free text boxes were included in the questionnaire where staff could expand on their answers. They were also asked for comments on how the study site could be improved. There were several comments from AL respondents indicating that they were not aware of the site. In response to this feedback, the study site will be included in staff development events, AL induction and embedded into general communications with ALs.

*How I was unaware of this site, I do not know. Now that I have discovered it, I am delighted to say, not only shall I refer to it now and again, but I shall also make a point of mentioning it to my students at the beginning of each new module session.*

*It all looks very useful, I just had not been aware of its existence before this survey, though I was aware of bits like the AYRF pages.*

In response to this feedback from SRF staff, there is also a need for including study site information into ongoing staff development sessions and induction.

*There is some good content here but it is not advertised/directed to Advisors—had it not been for this survey I would not have known this existed.*

*I wish I knew about this. As a relatively new member of staff, this will be a great resource for further learning for myself, and to direct learners to.*

All the responses from the SST when asked to provide any further information were positive. These included noting the advice forum as somewhere SST staff send students to when they ask about module choice advice.

*I regularly suggest students visit the forum for advice on module choices. Students tend to ask my advice, but I don’t have broad enough knowledge, so I direct them to that forum.*

*It is so valuable to us to have a resource like this to refer to and to steer students towards/provide a link. Student ‘self-serve’ is a big help and at least the more information they have can assist in their own decision-making process. That in turn can cut down any confusion and unnecessary information exchanges.*

*I like the ‘hub’ feel of this site. A go-to place for all M&S resources.*

## 5 Conclusion

Ensuring students are provided with appropriate academic, pastoral and social support is challenging. Providing such support for students studying online and at a distance can be further complicated by the isolation that students often experience when studying in this way. The move to online learning during the COVID-19 pandemic highlighted such challenges for several higher education institutions. The UK mathematics and statistics higher education community came together to share online techniques, tips and ideas through initiatives such as the Teaching and Learning Mathematics Online (TALMO, 2020) seminars and its associated website www.talmo.uk. The pandemic provided the opportunity to make greater use of resources such as videos, online quizzes and forums. However, creating online mechanisms for academic, pastoral and social support is not straightforward. The student experience for an online student is very different from that of a campus-based student. There was limited help available to institutions in the early stages of the pandemic to develop an online experience which would effectively support students at all stages of their journey from induction and orientation with the university through to completion of their studies and beyond.

During the pandemic mathematics and statistics departments have also grappled with ways to facilitate social interaction between students and between students and staff. This interaction is of paramount importance in learning, and therefore it is vital when designing online courses that the technology should meditate the type of collaboration which is necessary for learning mathematics (Borba & Llinares, 2012). Workshops were provided by the sigma network (www.sigma-network.ac.uk) in May 2020 to showcase systems and methods that could be used to support students during the pandemic with a follow up in May 2021 to share the lessons learnt from providing online mathematics and statistics support during the pandemic. Similar workshops were run by the Irish Mathematics Learning Support Network (www.imlsn.ie/index.php). Hodds (2020) reports that there was a drop in the number of students accessing mathematics support centres during the pandemic. One of several reasons given for the drop is a lack of advertising for the support or advertising in the wrong way, which is consistent with the findings in this study for the OU’s online learners. However, the way in which students were supported at the OU was relatively unchanged during the pandemic and the M&S study site with the embedded advice forum provided the online space to enable students studying a variety of M&S modules to connect with each other.

This paper has outlined how the School of M&S has endeavoured to deliver the student experience at a distance and online, by ensuring consistent academic, pastoral and social support is available to all students studying M&S modules. In order to assist staff that provide this support, a website, the M&S study site, has been created which all students studying M&S modules can engage and interact with. The study site follows each stage of the student lifecycle from first contact with the university to graduation and beyond. At each stage, the aim is for all units, both academic and non-academic who provide student support, to engage with the study site resources and use these to aid conversations with students. As this study has shown, not all ALs or SRF advisors are aware of the study site and more work needs to be done in terms of induction and ongoing staff development to rectify this. Ensuring that such resources form part of ongoing staff development sessions should be key to institutions who want to develop similar resources for use with their own students.

Since the creation of the M&S study site in 2017, there has been a steady increase in use. Evaluation of student usage suggests that students who discover the study site return to it on multiple occasions. However, there is an issue that only a fraction of the students who could use the site are aware of its existence. These findings echo those of a study into the online resources provided by the mathematics support centre at Maynooth University which showed that students who discovered the online resources found them useful, but there was an issue with advertising the resources to the wider student population (Mac an Bhaird *et al*., 2020). The findings from the M&S study site evaluation has resulted in the creation of a communication strategy to highlight the study site to all students studying the M&S modules, which took the form of a series of emails over the summer months. An ongoing communications strategy will be key to ensuring that all students are aware of the rich range of resources available.

One of the main impacts of the study site is the extent to which the SST staff use the site both to enhance their own knowledge about the curriculum and also directly in conversations with students. The study site not only helps ensure students are on the correct module, which is vital for retaining students, but also its maintenance and development have strengthened the link between the M&S SST and the School of M&S. It is only by the academic and support staff working closely together that students can access all forms of support in a coherent way.

As institutions are currently evaluating their own online provision, more attention needs to be given to the way in which the sector delivers all forms of support to students. Some of the online support outlined in this paper could be implemented when face-to-face teaching returns. There are advantages in having a subject-specific website which provides dedicated careers advice, module choice advice and resources to help students brush up on pre-requisite skills. Such sites are useful for both students who are specializing in that subject and those taking the subject as part of another qualification. However, for such a website to adequately support students, all members of staff who deliver support should be involved or at least be consulted in the construction and ongoing maintenance of the website. It is important that the website is clear and one way of helping students access the correct resources for the appropriate point in their lifecycle is for the sections to map onto the different student lifecycle stages. The M&S study site demonstrates one way of enabling academic and non-academic units to provide academic, pastoral and social support.

## Notes

**Rachel Hilliam** (Rachel.Hilliam@open.ac.uk) is a senior lecturer in statistics at the OU and a former director of teaching for the School of M&S.

## Notes

**Derek Goldrei** (Derek.Goldrei@open.ac.uk) FIMA is an honorary associate at the OU and an emeritus fellow of Mansfield College, Oxford. In his long career as an OU mathematics staff tutor and mathematics tutor at Oxford, he helped to develop and deliver methods of supporting mature students studying mathematics and statistics part-time and at a distance.

## Notes

**Gaynor Arrowsmith** (Gaynor.Arrowsmith@open.ac.uk) is the senior teaching manager in the School of M&S and is involved with many of the practicalities of teaching and learning in an open-entry distance institution. She has also been an AL in mathematics.

## Notes

**Alex Siddons** (Alexander.Siddons@open.ac.uk) is the operations manager of the M&S SST based in Manchester and oversees the provision of advice and guidance to students on mathematics modules and qualifications. He also oversees the provision of reasonable adjustments in exams for STEM students.

## Notes

**Cath Brown** (Cath.Brown@open.ac.uk) is an AL in the OU’s STEM faculty and former OU Students Association President.

## References

[ref1] de Carvalho Borba, M. & Linares, S. (2012) Online mathematics teaching education: overview of an emergent field of research. ZDM Math. Educ., 44, 697–704.

[ref2] Carlsen, A., Holmberg, C., Neghina, C. & Owusu-Buampong, A. (2016) Closing the gap: opportunities for distance education to benefit adult learners in higher education. UNESCO Institute for Lifelong Learning (UIL). Available at https://unesdoc.unesco.org/ark:/48223/pf0000243264 [date last accessed 18 June 2021].

[ref3] Calvert, C. & Hilliam, R. (2018) Student feedback to improved retention: using a mixed-methods approach to extend specific feedback to a generalisable concept. J. Open Distance Learn., 34, 103–117.

[ref4] Calvert, C., Hilliam, R. & Coleman, J. (2016) Improving retention for all students, studying mathematics as part of their chosen qualification, by using a voluntary diagnostic quiz. MSOR Connect., 14, 28–31.

[ref5] Diamond, A., Roberts, J., Vorley, T., Birkin, G., Evans, J., Sheen, J. & Nathwani, T. (2014) UK review of the provision of information about higher education: advisory study and literature review. Available at https://dera.ioe.ac.uk/19857/1/2014_infoadvisory.pdf [date last accessed 18 June 2021].

[ref6] Engineering Council . (2000) Measuring the mathematics problem*.* Available at https://www.engc.org.uk/engcdocuments/internet/Website/Measuring%20the%20Mathematic%20Problems.pdf [last accessed 18 June 2021].

[ref7] Foster, E., Lawther, S., Keenan, C., Bates, N., Colley, B. & Lefever, R. (2012) The HERE Project: Higher Education: Retention & Engagement*.* Available at https://s3.eu-west-2.amazonaws.com/assets.creode.advancehe-document-manager/documents/hea/private/resources/here_project_what_works_final_report_1568037216.pdf [last accessed 18 June 2021].

[ref8] Gallimore, M. & Stewart, J. (2014) Increasing the impact of mathematics support on aiding student transition in higher education. Teach. Math. Appl., 33, 98–109.

[ref9] Grove, M., Croft, T. & Lawson, D. (2020) The extent and uptake of mathematics support in higher education: results from the 2018 survey. Teach. Math. Appl., 39, 86–104.

[ref10] Hilliam, R (2020) The mathematics and statistics community of learners. eSTEeM the OU Centre for STEM pedagogy. Available at https://www.open.ac.uk/about/teaching-and-learning/esteem/sites/www.open.ac.uk.about.teaching-and-learning.esteem/files/files/2020-12-Rachel-Hilliam-final-report.pdf [date last accessed 18 June 2021].

[ref11] Hilliam, R. & Arrowsmith, G. (2019) Enhancing the student experience with the use of a dedicated subject website. MSOR Connect., 17, 39–45.

[ref12] Hilliam, R. & Goldrei, D. (2019) Creating an online mathematics and statistics community of learners. New Dir. Teach. Phys. Sci., 14. 10.29311/ndtps.v0i14.2824.

[ref13] Hilliam, R. & WIilliams, G. (2019) Academic and pastoral teams working in partnership to support distance learning students according to curriculum area. High. Educ. Pedagog., 4, 32–40.

[ref14] Hodds, M. (2020) A report into the changes in mathematics and statistics support practices due to Covid-19. Available at https://www.sigma-network.ac.uk/wp-content/uploads/2020/07/Report-into-the-changes-in-Maths-and-Stats-Support-practice-during-Covid-19.pdf [date last accessed 18 June 2021].

[ref15] Jooganah, K. & Williams, J. (2016) Contradictions between and within school and university activity systems helping to explain students’ difficulty with advanced mathematics. Teach. Math. Appl., 35, 159–171.

[ref16] Lawson, D. (2015) Mathematics support at the transition to university. Transitions in Undergraduate Mathematics Education (M. Grove, T. Croft, J. Kyle & D. Lawson eds). Birmingham: The Higher Education Academy, University of Birmingham, pp. 39–58.

[ref17] Lowe, T. (2015) Online quizzes for distance learning of mathematics. Teach. Math. Appl., 24, 138–148.

[ref18] Mac an Bhaird, C., Mulligan, P. & O’Malley, J. (2020) Mathematics support centre attendees and their use of online resources. MSOR Connect., 18, 63–69.

[ref19] McChlery, S. & Wilkie, J. (2009) Pastoral support to undergraduates in higher education. Int. J. Educ. Manag., 8, 23–36.

[ref20] Morgan, M. (2012) Improving the Student Experience: A Practical Guide of Universities and Colleges. Abingdon: Routledge.

[ref21] O’ Sullivan, C., Mac an Bhaird, C., Fitzmaurice, O. and Ní Fhloinn, E. (2014) An Irish mathematics learning support network (IMLSN) report on student evaluation of mathematics learning support: insights from a large scale multi-institutional survey*.* Available at https://www.sigma-network.ac.uk/wp-content/uploads/2019/02/IMLSN-Report-Student-evaluation-of-MLS-2016.pdf [date last accessed 18 June 2021].

[ref22] Pawley, S. & Hughes, C. (2018) Bridging the study gap: provision of support for mathematics students during breaks in study. New Dir. Teach. Phys. Sci., 13. 10.29311/ndtps.v0i13.2898.

[ref23] Pell, G. & Croft, A. (2008) Mathematics support—support for all? Teach. Math. Appl., 27, 167–173.

[ref24] Stodt, M. M. & Klepper, M. K. (1987) Increasing Retention: Academic and Student Affairs Administrators in Partnership (New Directions for Higher Education). San Francisco: Jossey-Bass.

[ref25] Soilemetzidis, I., Bennett, P., Buckley, A., Hillman, N. & Stoakes, G. (2014) The HEPI-HEA Student Academic Experience Survey 2014. Available at https://www.hepi.ac.uk/wp-content/uploads/2014/05/HEA_HEPI-Report_WEB_160514.pdf [date last accessed 1 June 2021].

[ref26] Talmo (2020) Teaching and learning mathematics online. Available at www.talmo.uk [date last accessed 18 June 2021].

[ref27] Temple, P., Callender, C., Grove, L. & Kersh, N. (2014) Managing the student experience in a shifting higher education landscape. The Higher Education Academy. Available at https://s3.eu-west-2.amazonaws.com/assets.creode.advancehe-document-manager/documents/hea/private/resources/managing_the_student_experience_1568037252.pdf [date last accessed 18 June 2021].

[ref28] The Open University . (2020) Facts and figures*.* Available at http://www.open.ac.uk/about/main/strategy-and-policies/facts-and-figures [date last accessed 18 June 2021].

[ref29] Warwick, J. (2010) Exploring student expectations in mathematics learning and support. Teach. Math. Appl., 29, 14–24.

[ref30] Weinbren, D. (2014) The Open University: A History. Manchester University Press, Manchester.

[ref31] Wilcox, P., Winn, S. & Fyvie-Gauld, M. (2006) ‘It was nothing to do with university it was just the people’: the role of social support in the first-year experience of higher education. Stud. High. Educ., 30, 707–722.

